# Insidious Onset of Incarcerated Parastomal Hernia With Gastric Outlet Obstruction: A Case Report

**DOI:** 10.7759/cureus.26930

**Published:** 2022-07-17

**Authors:** Hassan Baig, Heather Curry, Christopher P Leiberman, Ahmed M Al Ani, Carol M Watson

**Affiliations:** 1 Department of General Surgery, Queen Elizabeth University Hospital, Glasgow, GBR; 2 Department of Surgery, Queen Elizabeth University Hospital, Glasgow, GBR

**Keywords:** colorectal surgery, gastric outlet obstruction, incarcerated hernia, general surgery, gastric parastomal hernia

## Abstract

Although parastomal hernias have a high incidence in the general population, involvement of the stomach remains rare due to the numerous suspensory structures tethering this organ in its anatomical location. This case details a 75-year-old lady with a painless onset of a gastric parastomal hernia with progressive incarceration over a two-week period. The deteriorating clinical condition of the patient following weeks of stability indicated that the cause of symptoms is likely sinister. Imaging confirmed incarceration of the stomach within a parastomal hernia. Although this has been reported previously, there is little to suggest this condition exists with an insidious onset. Patients who are at high risk of gastric herniation and who fit this clinical vignette with a known parastomal hernia should be offered prompt investigations to ascertain the diagnosis and facilitate further management.

## Introduction

Parastomal hernias (PH) are defined as an abnormal herniation of tissue or a solid abdominal organ through the deliberately formed fascial defect of the stoma site. The herniation occurs due to the ongoing forces against the anterior abdominal wall from the abdominal visceral organs. Most commonly, PHs involve omental or peritoneal fat or small or large bowel segments. Each of these structures has the potential to become incarcerated and/or strangulated.

Numerous risk factors are associated with patients developing PHs, with obesity being the leading factor. Additional risk factors include increasing age, poor nutritional status, corticosteroids, and surgical technique [1]. Herniation of the stomach is an uncommon finding through parastomal herniae and can lead to symptoms representing gastric outlet obstruction [2]. A gastric hernia can subsequently become incarcerated or strangulated representing a surgical emergency [3].

Reaching this diagnosis can prove difficult based on clinical history, examination, and biochemical results alone due to the rarity of this condition. Patients would exhibit signs typical of gastric outlet obstruction such as nausea, vomiting, and metabolic alkalosis with respiratory compensation. However, for a definitive diagnosis of the cause of gastric outlet obstruction, cross-sectional imaging is required with computed tomography (CT) being the investigation of choice. Oesophagogastroduodenoscopy can also be carried out for diagnostic purposes to rule out any other cause for symptoms [1].

Gastric parastomal hernias are managed initially through gastric decompression by nasogastric tube, and analgesia, adopting a conservative approach. However, owing to patient fitness and stability, an operative procedure is the corrective intervention, through the removal of the stomach from the hernial sac, and closure of the defect in the abdominal wall [4].

## Case presentation

A 75-year-old female patient with a previous history of emergency sigmoidectomy for perforated sigmoid diverticulum presented with a 2-week history of vomiting, nausea, and lack of appetite. Additional past medical history included heart failure, atrial fibrillation, recurrent coliform urinary tract infections, chronic obstructive pulmonary disease, chronic kidney disease stage 3, and ex-smoker. The patient had an end colostomy site in the left iliac fossa following a previous Hartmann’s procedure. Three days prior to general surgical referral, the patient developed coffee ground vomiting, pathognomonic with an upper gastrointestinal bleed.

**Figure 1:**
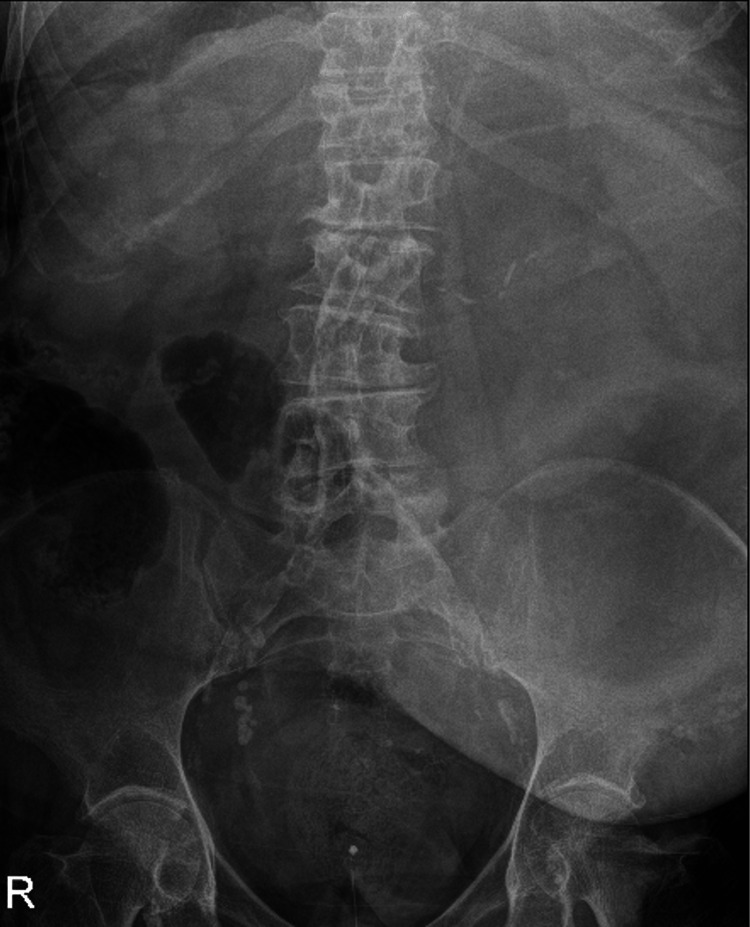
Abdominal X Ray showing no dilated viscera

The 2-week history of vomiting was without any abdominal pain but the patient had a new oxygen requirement and metabolic alkalosis. She initially had a computed tomography pulmonary angiogram (CTPA) which did not reveal any abnormality within the upper abdominal organs, within the limits of the CTPA study. A parastomal hernia was evident on examination intermittently causing pain, which was a longstanding symptom. Coffee ground vomit was noted by the parent team, combined with a haemoglobin drop and raised urea this was managed as a suspected upper gastrointestinal bleed and the patient was listed for gastroscopy. The colostomy output was unchanged with no evidence of melaena. An abdominal X-ray was performed which did not show dilated viscera (Figure 1). The initial surgical review noted a non-distended abdomen with a soft and minimally tender parastomal hernia and recommended computed tomography of the abdomen and pelvis for further assessment (CTAP). The hernia could not be manually reduced due to the size and discomfort to the patient.

The patient’s observations revealed a respiratory rate of 26 breaths per minute, saturation of 97% on 2 litres of supplemental oxygen, a heart rate of 118 beats per minute, and blood pressure of 94/59 mmHg. At the time of review, there was a discussion with the patient and relatives that this patient was not fit for any surgical intervention due to extensive co-morbidities.

The patient’s biochemistry results revealed progressively worsening renal function and hypokalaemia which was treated with potassium replacement therapy. On the day of the initial coffee-ground vomit, the patient’s blood results were as demonstrated in Table 1. Whilst waiting for the cross-sectional imaging, there was a significant deterioration in the biochemical parameters of the patient (Table 1). 

**Table 1:** Initial and deteriorating blood tests with respective results mmol/L - millimoles per litre umol/L - micromoles per litre ml/minute - millilitres per minute mg/L - milligrams per litre x10^9^/L - billion white blood cells per litre

Blood Test	Initial Blood Results	Blood results at Deterioration
Urea	10.9 mmol/L	20.6 mmol/L
Creatinine	104 umol/L	199 u​​​​​​​mol/L
Estimated Glomerular Filtration Rate	45 ml/minute	21 ml/minute
C-Reactive Protein	34 mg/L	200 mg/L
White Blood Cell Count	18.4 x10^9^/L	12.1 x10^9^/L

**Figure 2:**
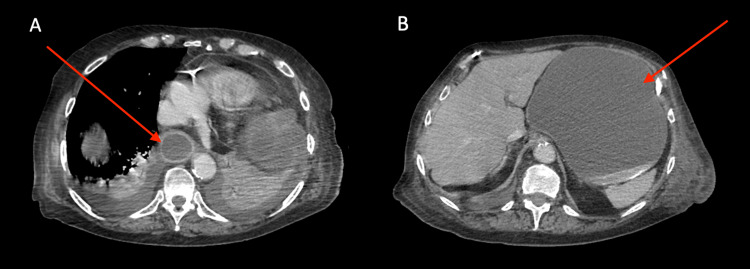
Computed Tomography of the Abdomen and Pelvis revealing distended viscera A - Dilated, fluid-filled distal oesophagus B - Dilated, fluid-filled stomach

**Figure 3:**
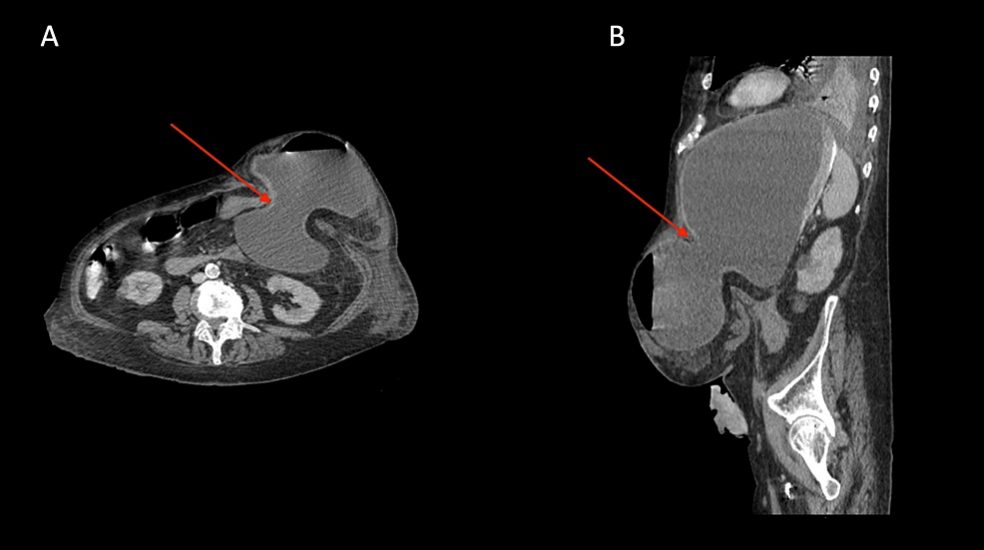
Computed tomography of abdomen and pelvis revealing efferent loop at the neck of the hernia A - Axial view of pylorus emerging as an efferent loop at the hernial neck B - Sagittal view of pylorus emerging as an efferent loop at the hernial neck

A CTAP was subsequently conducted. The imaging revealed a gastric outlet obstruction secondary to partial prolapse of the body of the stomach into the parastomal hernia. There was clear upstream dilatation of the gastric fundus and evidence of a fluid-filled, distended lower oesophagus (Figure 2). There was a visible transition point at the pyloric antrum which emerged as an efferent loop at the neck of the hernia (see Figure 3). The gastric wall showed patchy enhancement raising concerns regarding its integrity. However, there was no clear gastric perforation. There was clear layering of a high-density material posteriorly within the fundus likely representing blood or ingested material. There was no evidence of free fluid within the hernial sac which also contains several collapsed loops of the transverse colon continuous with the end colostomy.

Upon repeat assessment of the patient by the general surgical team and having taken into consideration the numerous co-morbidities, a conservative approach was taken to manage this critically unwell patient. Decompression of the stomach with NG was advised and provided a degree of symptomatic relief. The incarceration of the stomach within the parastomal hernia left few choices for management, particularly given the current situation of the patient and the likelihood of her not surviving general anaesthesia.

Despite the best conservative management and efforts, the patient was unable to survive this condition and unfortunately passed away on the day of referral to the surgical team.

## Discussion

PHs are a common complication associated with stoma-forming surgeries. The incidence of this complication has been reported as up to 48% [5]. Generally, PHs contain pre-peritoneal fat, intraperitoneal fat or contain segments of the bowel. PH containing the stomach is an extremely rare presentation with just 20 cases reported upon review of the current literature. Almost all the cases of gastric herniation were through a colostomy site, with this condition affecting females >75 years of age. Additional factors which increase the risk of parastomal herniation include increasing age, obesity, infection, smoking, and chronic obstructive pulmonary disease. In this patient, there was a strong history of tobacco abuse, prior diagnosis of COPD, female sex, and formation of an end colostomy [3]. These factors increase the combined risk of developing a PH, and thereby a gastric PH.

The anatomy of the stomach, meaning its fixed position by suspensory ligaments, such as the hepatogastric, gastrophrenic and gastrosplenic ligaments, and tethering blood supply prevents the stomach from having much mobility at all [4]. The surrounding structures and organs alongside these ligaments provide integrity and structural support thereby reducing laxity and effectively preventing gastric herniation. In the cases of gastric PH, these ligaments have often loosened over time in combination with a fascial defect from the stoma site, thereby allowing the stomach to slip into the hernia sac.

PHs are mostly asymptomatic, however, they carry a risk of incarceration and/or strangulation which can significantly increase morbidity and mortality. The initial diagnosis is primarily clinical with thorough history and examination, and careful examination of the stoma with both digital internal and external examination. Almost all the reported cases of GPH report a degree of gastric outlet obstruction, with most cases reporting a complete obstruction leading to hyperemesis, which is the primary presenting complaint alongside abdominal pain, nausea, bloating and abdominal distension [1]. Diagnosis should be confirmed through cross-sectional imaging with computed tomography as the imaging of choice given the speed of acquisition, and the potential of administering oral contrast to assess whether any perforation is evident [6]. In patients with incarceration, oesophagogastroduodenoscopy (OGD) should be avoided as there is a high risk of perforation, particularly due to air insufflation, and it provides little therapeutic benefit.

In the case of this patient, she had a prolonged presentation of symptoms, and given the rarity of this type of herniation, her diagnosis was not clear until a later stage. Given the length of the presentation, it is likely that initially a degree of gastric outlet obstruction was observed, which over time progressed to a complete gastric outlet obstruction due to herniation of the pylorus through the parastomal hernia defect. This herniation likely led to inflammation and swelling of the stomach, thereby causing incarceration as seen in the cross-sectional imaging, with surrounding fat stranding. Unfortunately, due to the comorbidities of this patient, coupled with the progression of her condition, she was deemed unfit to survive major abdominal surgery. Conservative management was attempted but was also unsuccessful in improving her condition.

Importantly, gastric parastomal hernias must be recognised early to achieve optimal outcomes through good clinical history taking and examination. CT imaging should be the imaging of choice (+/- oral contrast), and invasive investigations such as OGD should be avoided unless there is a therapeutic element to them so as not to, theoretically, cause further complications. Depending on the degree of the herniation, confirmation of the diagnosis should prompt an initially conservative approach through nasogastric tube decompression, manual reduction of the hernia, and the use of a hernia truss. Failing a conservative approach, operative management should be offered, owing to the patient being a suitable candidate for this with the repair of the hernial defect.

## Conclusions

This study emphasises the importance of recognising the rare presentation of GPH. It must be recognised that this is a condition primarily affecting females with end colostomies. Through the literature review, there is a higher risk of developing GPH with female sex, increasing age, previous diagnosis of COPD and previous end colostomy formation. The spectrum of presentations should be recognisable by clinicians prompting a swift response by initiating cross-sectional imaging and a conservative approach. Invasive diagnostic tests such as OGD should be avoided given the high diagnostic sensitivity of CT imaging in parastomal hernias. Operative intervention should be the final step of the management pathway offered in an emergency if there is evidence of incarceration, strangulation or perforation, and electively if the symptoms are recurring or worsening.

## References

[1] Eastment J, Burstow M (2018). Parastomal stomach herniation complicated by gastric outlet obstruction: a case report and literature review. Int J Surg Case Rep.

[2] Anandan M, Roberts-Thomson J, Lynch C (2020). Gastric outlet obstruction due to a parastomal hernia: case report of a robotic-assisted laparoscopic surgery and literature review. ANZ J Surg.

[3] Khan S, Khan M, Harris M, Murphy SR, Dionisio P (2022). A case of gastric outlet obstruction secondary to parastomal stomach herniation. Cureus.

[4] Ahmed A, Dana S, Hisham E, Malek H, Rudolf Z (2020). Incarcerated stomach in a parastomal hernia. Ann African Surg.

[5] Johnson K, Monroe N, Protyniak B (2020). The other double bubble sign: gastric parastomal hernia. CRSLS.

[6] Bota E, Shaikh I, Fernandes R, Doughan S (2012). Stomach in a parastomal hernia: uncommon presentation. BMJ Case Rep.

